# Cannabidiol treatment improves metabolic profile and decreases hypothalamic inflammation caused by maternal obesity

**DOI:** 10.3389/fnut.2023.1150189

**Published:** 2023-03-09

**Authors:** Fernanda da Silva Rodrigues, Jeferson Jantsch, Gabriel de Farias Fraga, Victor Silva Dias, Sarah Eller, Tiago Franco De Oliveira, Márcia Giovenardi, Renata Padilha Guedes

**Affiliations:** ^1^Graduate Program in Biosciences, Federal University of Health Sciences of Porto Alegre (UFCSPA), Porto Alegre, Rio Grande do Sul, Brazil; ^2^Undergraduate Program in Biomedical Sciences, Federal University of Health Sciences of Porto Alegre (UFCSPA), Porto Alegre, Rio Grande do Sul, Brazil; ^3^Graduate Program in Health Sciences, Federal University of Health Sciences of Porto Alegre (UFCSPA), Porto Alegre, Rio Grande do Sul, Brazil

**Keywords:** maternal obesity, neuroinflammation, cannabidiol, hypothalamus, insulin resistance

## Abstract

**Introduction:**

The implications of maternal overnutrition on offspring metabolic and neuroimmune development are well-known. Increasing evidence now suggests that maternal obesity and poor dietary habits during pregnancy and lactation can increase the risk of central and peripheral metabolic dysregulation in the offspring, but the mechanisms are not sufficiently established. Furthermore, despite many studies addressing preventive measures targeted at the mother, very few propose practical approaches to treat the damages when they are already installed.

**Methods:**

Here we investigated the potential of cannabidiol (CBD) treatment to attenuate the effects of maternal obesity induced by a cafeteria diet on hypothalamic inflammation and the peripheral metabolic profile of the offspring in Wistar rats.

**Results:**

We have observed that maternal obesity induced a range of metabolic imbalances in the offspring in a sex-dependant manner, with higher deposition of visceral white adipose tissue, increased plasma fasting glucose and lipopolysaccharides (LPS) levels in both sexes, but the increase in serum cholesterol and triglycerides only occurred in females, while the increase in plasma insulin and the homeostatic model assessment index (HOMA-IR) was only observed in male offspring. We also found an overexpression of the pro-inflammatory cytokines tumor necrosis factor-alpha (TNFα), interleukin (IL) 6, and interleukin (IL) 1β in the hypothalamus, a trademark of neuroinflammation. Interestingly, the expression of GFAP, a marker for astrogliosis, was reduced in the offspring of obese mothers, indicating an adaptive mechanism to *in utero* neuroinflammation. Treatment with 50 mg/kg CBD oil by oral gavage was able to reduce white adipose tissue and revert insulin resistance in males, reduce plasma triglycerides in females, and attenuate plasma LPS levels and overexpression of TNFα and IL6 in the hypothalamus of both sexes.

**Discussion:**

Together, these results indicate an intricate interplay between peripheral and central counterparts in both the pathogenicity of maternal obesity and the therapeutic effects of CBD. In this context, the impairment of internal hypothalamic circuitry caused by neuroinflammation runs in tandem with the disruptions of important metabolic processes, which can be attenuated by CBD treatment in both ends.

## 1. Introduction

Maternal malnourishment before and during pregnancy is a growing worldwide concern known to bear several implications for fetal development that lead to long-term consequences on offspring health and well-being (1, 2). Interestingly, since the pioneering studies on the effects of the perinatal environment, such as the Developmental Origin of Health and Disease (DOHaD) theory, the context transitioned from the lack of nutrients due to hunger and starvation to excess due to the global obesity pandemic (3). In fact, globalization and urbanization have gradually led to an increase in the consumption of “junk food” (i.e., ultra-processed, rich in fats, and sugar) associated with the reduction of physical activity, a phenomenon called “nutritional transition” (4, 5). In this context, it is important to highlight that obesity and the consumption of mentioned “junk food” most often co-occur, making it difficult to discriminate the effects of obesity and its metabolic profile *per se* from those related to nutritional aspects of the foods consumed (6).

A broad range of studies has demonstrated that an abnormal inflammatory milieu during *in utero* development triggers the so-called “early-life programming” of the offspring metabolism (3, 7). A number of potential pathways underlying the effects of maternal obesity include increased sustained inflammation, changes in lipid transport and storage, dysregulation of glucose metabolism, and modifications to the microbiome, which triggers increased translocation of lipopolysaccharides (LPS) to the bloodstream and circulating levels of pro-inflammatory cytokines (8–10). These inflammatory mediators are able to cross the placental barrier and create a harmful environment for developing fetal tissues (11, 12). Other than that, the increased insulin resistance, glucose levels, and lipids, with a potentially elevated supply of nutrients to the developing fetus, contribute to setting persistent changes in the offspring’s energy balance, appetite regulation, lipid and glucose homeostasis, and gut dysbiosis. Overall, as a result, maternal obesity substantially raises the risk of offspring obesity, insulin resistance, type 2 diabetes, high blood pressure, and adverse lipid profile (7, 10, 13).

The hypothalamus is the predominant brain area that controls energy balance by integrating information from the body and initiating appropriate behavioral, humoral, and neural outputs. Current evidence indicates hypothalamic inflammation as a likely mechanism for the dysregulation of the homeostatic control of energy balance, which might lead to an increased susceptibility to metabolic alterations and obesity in the offspring (14, 15). Abnormal insulin signaling during neurodevelopment leads to malformation of neural projections that affect hypothalamic function and plasticity, resulting in altered energy homeostasis in the offspring (16, 17). These effects can also be attributed to enhanced activation of resident immune cells, such as astrocytes and microglia, with the consequent secretion of inflammatory cytokines, such as tumor necrosis factor-alpha (TNFα), interleukin (IL)-6, and IL-1β (18).

The extent to which changes in the offspring’s habits and resolutive approaches throughout life can modify the effects of perinatal maternal obesity is yet not known, but a few anti-inflammatory approaches seem to exert positive effects. The endocannabinoid system has been extensively studied in the context of obesity and inflammation, showing a close relationship with energy metabolism and the feeding circuitry (19). Cannabidiol (CBD) is a non-psychotropic terpenophenol isolated from *Cannabis sativa* with anti-inflammatory and antioxidant effects discussed to be beneficial for diverse immunological states (20). It has been suggested that the hydroxyl groups of the phenol ring in CBD structure interfere with free radical chain reactions, which confers CBD its antioxidant activity (20, 21). Furthermore, the modulation of endocannabinoid signaling *via* downregulation of CB1 receptor activity and upregulation of CB2 receptor activity results in reduced reactive oxygen species (ROS) production and reduced pro-inflammatory signaling (22, 23).

Besides the endocannabinoid receptors CB1 and CB2, CBD is also known to interact with other systems and receptors relevant to metabolic homeostasis, such as the G protein-coupled receptor 55 (GPR55), Transient Receptor Potential Vanilloid (TRVP) channel and nuclear peroxisome proliferator-activated receptors (PPARs) (24–27). This broad spectrum of communication among systems translates into modulatory roles in diverse metabolic aspects throughout the entire body, from lipid metabolism and storage in the liver to mitochondrial activity and energy expenditure in the adipose tissue (28, 29). Furthermore, CBD was also shown to interact with glucose metabolism by improving glucose tolerance (30, 31), the brain-gut axis by mitigating microbiome dysbiosis (30, 32, 33), and hypothalamic anorexigenic neuromodulators (34). In this sense, beyond directly improving a range of physiological aspects related to obesity, modulation of the endocannabinoid system seems to also be effective on tempering eating behaviors, such as high-fat and high-sucrose food intake (35), hyperphagia (36), sucrose self-administration (37) and binge eating (38), which sets the stage for it as a potential intervention on maternal obesity-related metabolic dysfunctions.

Here, we investigated the effects of CBD treatment on maternal obesity-induced hypothalamic inflammation and metabolic outcomes on the early-adulthood of the offspring.

## 2. Materials and methods

### 2.1. Animals

Eighteen female Wistar rats (3-weeks-old) were obtained from the animal facility of the Federal University of Health Sciences of Porto Alegre (UFCSPA). The animals were group-housed (3 animals per cage) under standard laboratory conditions at a controlled temperature (23 ± 1°C) and 12-h light:dark cycle. This study was approved by UFCSPA Institutional Animal Care and Use Committee under protocol N° 751/21. All experiments were designed and performed to minimize the number and suffering of subjects, following the international laws that regulate the care of laboratory animals.

### 2.2. Experimental groups and diet

Three-week-old female breeders (*N* = 9) were placed on either control diet (CT) composed by standard chow (Nuvilab^®^ CR-1 Nuvital^®^, Curitiba, PR, Brazil) (3.4 kcal/g, 63% carbohydrates, 26% protein, and 11% fat) or a Cafeteria Diet (CAF) composed by standard chow plus bacon mortadella (Perdigão^®^), strawberry wafers (Isabela^®^), chocolate cookies (Isabela^®^), pizza-flavored crackers (Parati^®^), white chocolate (Harald^®^), sausage (Alibem^®^), and orange-flavored soda (Sukita^®^) (4.3 kcal/g, 43% carbohydrates, 14% protein, and 43% fat) with water *ad libitum*. CAF group was fed with three menus with different combinations among the foods mentioned interchanged every 2 days, to maintain novelty and stimulate consumption. CAF was chosen as an obesogenic diet since it mimics the Western dietary habits in a more translational manner than regular high-fat and/or high-sugar diets, once CAF provides the variety of textures, options and palatability that contribute to hedonic eating and are not present on manufactured chows (39, 40). The diets were maintained for 12 weeks prior to and during mating with a 3-months-old male, throughout gestation, lactation, and until weaning. Dam weight and the weight of consumed diets were recorded weekly. Day of parturition was considered postnatal day zero (PND0).

To reduce the impact of litter effects, litters were adjusted to seven to nine pups per dam with an equal proportion of males to females when possible. All offspring were weaned at PND21, placed on standard chow and weighed weekly. Litters were divided equally among treatment groups and by sex, which created four groups per sex: CT mother + Vehicle (CT-Veh), CT mother + Cannabidiol (CT-CBD), CAF mother + Vehicle (CAF-Veh), and CAF mother + Cannabidiol (CAF-CBD).

**FIGURE 1:**
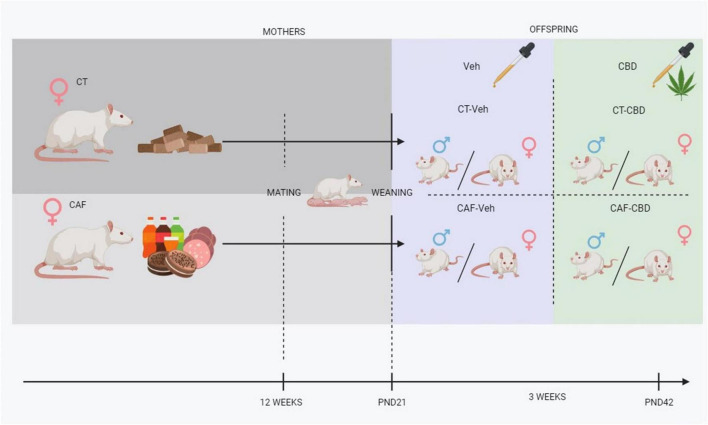
Experimental design. CT, control chow-fed dam; CAF, cafeteria diet-fed dam; Veh, offspring treated with vehicle (corn oil); CBD, offspring treated with cannabidiol (50 mg/kg); PND, post-natal day.

Treatment started at the same day of weaning (PND21) and consisted of CBD oil diluted in corn oil for a dose of 50 mg/kg (Prati-Donaduzzi^®^, Toledo, PR, Brazil) or corn oil (vehicle), both in a volume of 1 mL/kg by oral gavage. The treatment was administered 7 days a week for 3 weeks (Figure 1). Treatment dose and duration were chosen based on previous studies on different cognitive-assessment models with oral administration of CBD (41–45). Furthermore, we have performed a pilot study assessing doses of 2,5 mg/kg, 10 mg/kg, and 50 mg/kg, to which 50 mg/kg showed most significant positive results (data not shown).

### 2.3. Tissue processing

At the end of the 3 weeks of treatment, on PND42, animals were euthanized by decapitation. The gonadal visceral adipose tissue was weighed, truncal blood was centrifuged, and plasma was separated. The brain was dissected immediately and all tissues were snap-frozen in liquid nitrogen and stored in −80°C for further processing and analysis.

### 2.4. Biochemical analysis

Fasting plasma levels of glucose, total cholesterol and triglycerides were quantified using enzymatic colorimetric kits (Labtest, Lagoa Santa, Brazil). Insulin levels in the plasma were determined by enzyme-linked immunosorbent assay (ELISA) (Insulin ELISA kit, Cat# RAB0904; Sigma-Aldrich, St. Louis, MO, USA). Subsequently, the homeostatic model assessment (HOMA-IR) index was calculated to determine insulin resistance through the following formula: glucose (mg/dL) × insulin (uU/mL)/22.5.

### 2.5. LPS quantification

Plasma (150 μL) was hydrolyzed with 75 μL of NaCl 150 mM and 300 μL of HCl 8M and then incubated for 4 h at 90°C. Afterward, 3 mL of hexane were added, and samples were centrifuged at 3,500 rpm for 10 min. The upper organic phase was withdrawn, and the residue was reconstituted in 50 μL of methanol, transferred to a vial, and an aliquot of 3 μL was injected into the analytical system. A Nexera-i LC-2040C Plus system coupled to a LCMS-8045 triple quadrupole mass spectrometer (Shimadzu, Kyoto, Japan) was used for the analysis.

### 2.6. RT-qPCR

Total RNA was isolated from the hypothalamus using TRIzol^®^ (Invitrogen, Brazil) according to the manufacturer’s instructions. The quantification of total RNA was done by spectrometry BioSpec-nano^®^ (Shimazu, Kioto, Japan) at 260 and 280 nm. For cDNA synthesis, 1,000 ng of RNA were reverse transcribed according to the manufacturer’s instructions (GoScript Reverse Transcription Kit, Promega, Brazil). To conduct real time quantitative polymerase chain reaction (RT-qPCR), cDNA was added to a reaction mix (10 μL final volume) containing 100 nM gene-specific primers and universal SYBR green supermix (Applied Biosystems, Thermo Fisher Scientific CA, USA). All samples were run in duplicate and were analyzed on an QuantStudio Real-Time PCR instrument (Applied Biosystems, Thermo Fisher Scientific CA, USA) for quantitative monitoring of PCR product formation. Relative gene expression was normalized to β-Actin controls and assessed using the 2-ΔΔCT method. Primer sequences are as follows: β-Actin: F: TATGCCAACACAGTGCTGTCTGG; β-Actin: R: TACTCCTGCTTGCTGATCCACAT; Iba1: F: GCAAG GATTTGCAGGGAGGA; Iba1: R: CGTCTTGAAGGCCTCCAG TT; GFAP: F: CGAAGAAAACCGCATCACCA; GFAP: R: CC GCATCTCCACCGTCTTTA; TNFα: F: TGGCGTGTTCATCCG TTCTCTACC; TNFα: R: CCCGCAATCCAGGCCACTACTT; IL6: F: GACCAAGACCATCCAACTCATC; IL6: R: GCTTAG GCATAGCACACTAGG; IL1β: F: TGAGGCTGACAGACCCCAA AAGAT; IL1β: R: GCTCCACGGGCAAGACATAGGTAG.

### 2.7. Data analysis and statistics

Data were analyzed using Graphpad Prism 9 statistical software (GraphPad Software, San Diego, CA, USA). Two-way ANOVA with a Bonferroni *post hoc* analysis was performed within sexes. The main effects were: maternal diet and CBD treatment. The interaction between these two factors was also analyzed. The results were expressed as the mean ± standard error of the mean (SEM). Outliers were removed using the ROUT test, and statistically significant differences were considered at *p* < 0.05.

## 3. Results

### 3.1. Cafeteria diet induces obesity in female Wistar rats after 9 weeks of diet

**FIGURE 2:**
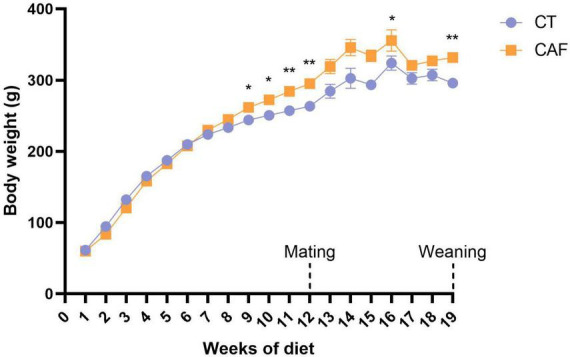
Dams’ body weight throughout the experiment. Cafeteria diet-fed (CAF) dams presented significantly higher body weight when compared to control diet (CT) from the 9th week of diet, which was consistent until mating (12th week of diet) and after weaning of the offspring (19th week of diet). Data are presented as mean ± SEM. *n* = 9/group. *p < 0.05 **p < 0.01.

The dams from both CT and CAF groups were weighed every week throughout the experiment to assess the impact of the diets on weight gain. Repeated measures two-way ANOVA has shown a significant diet effect (*F*_1,16_ = 8.705; *p* = 0.0094). From the 9th week of diet, CAF-fed female Wistar rats presented significantly higher body weight than the CT group (*p* = 0.0349), which persisted until mating in the 12th week (*p* = 0.0062). Despite no differences in body weight being shown during most of gestational and lactational time, except for the 15th week (*p* = 0.0298), the difference became significant again right after weaning of the offspring on the 19th week (*p* = 0.0012) (Figure 2).

### 3.2. Cafeteria-induced maternal obesity increases visceral white adipose tissue deposits even though it does not affect offspring total body weight

**FIGURE 3:**
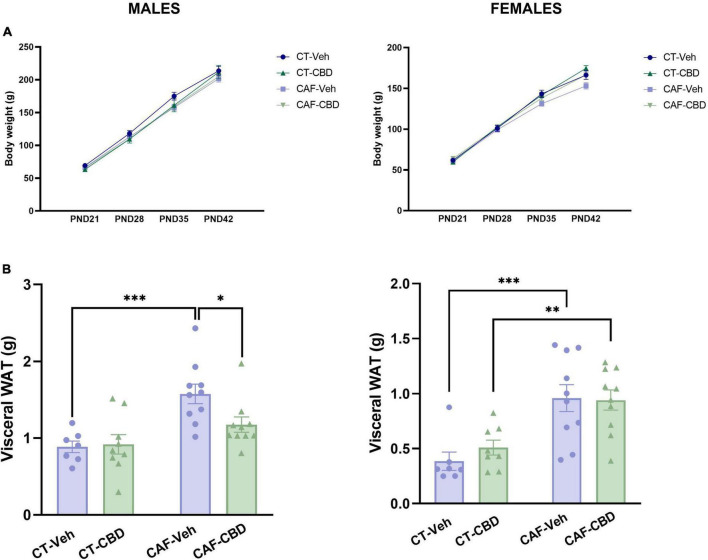
Offspring’s body weight and visceral white adipose tissue (WAT). Neither male nor female offspring showed influences of maternal diet or cannabidiol (CBD) treatment in weight at weaning (PND21) and the following 3 weeks of treatment **(A)**. Maternal diet increased visceral fat deposit in both male and female cafeteria diet (CAF)-Veh offspring with CBD effect only in males **(B)**. Data are presented as mean ± SEM. *n* = 8–10/group. *p < 0.05 ^**^p < 0.01 ^***^p < 0.001.

The offspring was weighed weekly from weaning (PND21) to euthanasia (PND42) to determine weight gain in early-life and throughout treatment and visceral white adipose tissue (WAT) was weighed at euthanasia. No groups showed any differences in weight gain related to neither maternal diet nor CBD treatment (Figure 3A), however, both male and female offspring of CAF-fed dams, presented an increase in WAT (maternal diet effect: *F*_1,32_ = 17.02; *p* = 0.0002 and *F*_1,31_ = 24.92; *p* < 0.0001 respectively). Indeed, untreated male offspring of obese dams (CAF-Veh) showed heavier visceral WAT when compared to the offspring of control dams (CT-Veh) (*p* = 0.0005). Also, both female CAF-Veh and CAF-CBD had more visceral fat than CT ones (*p* = 0.0008 and *p* = 0.0084). However, CBD treatment was able to reduce the deposition of visceral fat on male CAF-CBD when compared to CAF-Veh (*p* = 0.0256) (Figure 3B).

These data suggest a complex energy-balance disruption on the offspring of obese mothers, with an increase in the accumulation of visceral fat while maintaining total body weight. Also, CBD treatment seems to exert a positive effect in a sex-dependent manner.

### 3.3. Female offspring lipid profile is more affected by CAF-induced maternal obesity with partial effects of cannabidiol treatment

**FIGURE 4:**
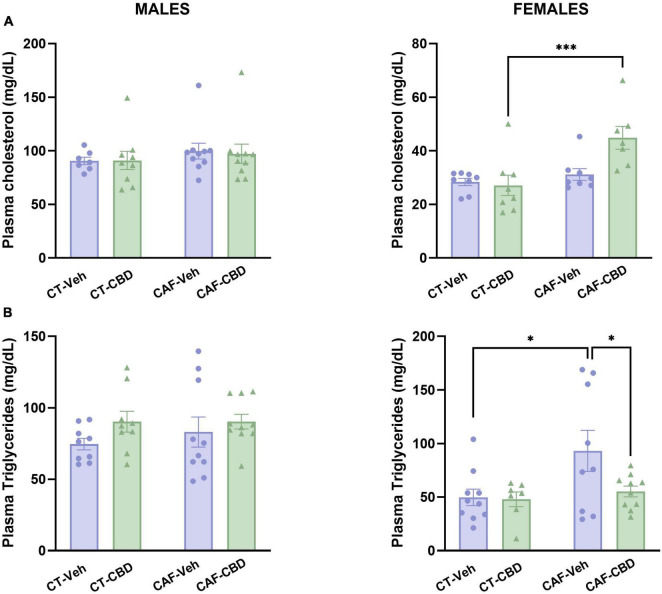
Offspring’s plasma levels of total cholesterol and triglycerides. Female cafeteria diet (CAF)-cannabidiol (CBD) presented higher levels of plasma cholesterol than control diet (CT)-CBD **(A)**. Maternal diet increased triglyceride levels in females, but CBD treatment was able to revert this effect **(B)**. No differences were seen in males. Data are presented as mean ± SEM. *n* = 8–10/group. *p < 0.05 ^***^p < 0.001.

Plasma cholesterol and triglyceride levels were assessed in order to evaluate the biochemical profile of the offspring. On female offspring, there was a maternal diet effect (*F*_1,31_ = 11.29; *p* = 0.0023) and an interaction between diet and CBD treatment (*F*_1,31_ = 6.027; *p* = 0.0208) on total cholesterol levels. CAF-CBD had higher levels of plasma cholesterol than CT-CBD (*P* = 0.0008) (Figure 4A). Regarding triglycerides, there was a maternal diet effect (*F*_1,32_ = 4.997; *p* = 0.0325). CAF-Veh presented higher levels of triglycerides when compared to CT-Veh (*P* = 0.0166), while CAF-CBD showed lower levels when compared to CAF-Veh (*P* = 0.0395) (Figure 4B). There were no significant differences among male groups.

Together, these data suggest that CAF-induced maternal obesity affects lipid metabolism in the offspring in a sex dependent manner, with apparently more severe effects in females. However, even though CBD treatment did not exert any effects on total cholesterol, it was able to revert the increased triglyceride levels in females.

### 3.4. Cannabidiol treatment reverts insulin resistance caused by maternal obesity in male offspring

**FIGURE 5:**
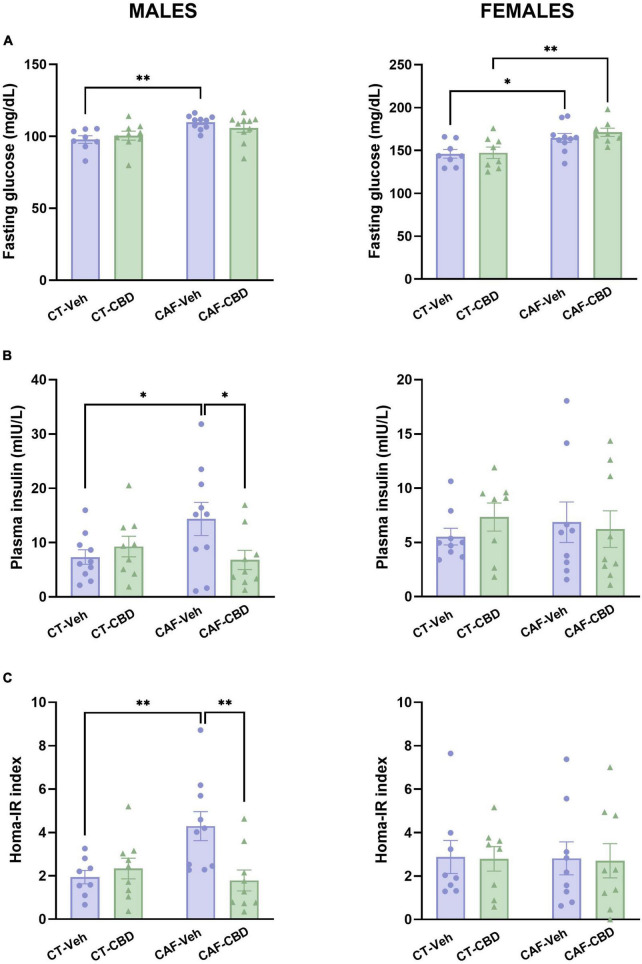
Plasma levels of glucose and insulin and calculated homeostatic model assessment (HOMA-IR) index of the offspring. Maternal obesity increased fasting glucose levels in both male and female offspring with no cannabidiol (CBD) effect **(A)**. Maternal obesity increased levels of plasma insulin in males, but CBD treatment was able to revert this damage **(B)**. Male cafeteria (CAF)-vehicle (Veh) presented an elevated HOMA-IR index, which was alleviated by CBD treatment **(C)**. Data are presented as mean ± SEM. *n* = 8–10/group. *p < 0.05 ^***^p < 0.01.

Plasma levels of fasting glucose and insulin were evaluated, and the HOMA-IR was determined in order to assess glucose metabolism and insulin resistance in the offspring. In male offspring we found a maternal diet effect on glucose levels (*F*_1,33_ = 10.60; *p* = 0.0026). CAF-Veh had higher fasting glucose than CT-Veh (*p* = 0.0072), with no effect of CBD (Figure 5A). On insulin, there was an interaction between maternal diet and CBD treatment (*F*_1,34_ = 4.916; *p* = 0.0334). CAF-Veh showed higher insulin levels than CT-Veh (*p* = 0.0458), while CAF-CBD had lower insulin levels than CAF-Veh (*p* = 0.0354) (Figure 5B). Consequently, on the HOMA-IR there was an interaction between maternal diet and CBD treatment (*F*_1,32_ = 7.674; *p* = 0.0093). CAF-Veh showed an increased HOMA-IR when compared to CT-Veh (*p* = 0.0068), while CAF-CBD had a lower index than CAF-Veh (*p* = 0.0029) (Figure 5C).

In female offspring, fasting glucose levels showed a maternal diet effect (*F*_1,30_ = 15.30; *p* = 0.0005). Both female CAF-Veh and CAF-CBD showed higher levels of plasma glucose when compared to their CT (*p* = 0.0377 and *p* = 0.0099) (Figure 5A). However, we did not find differences regarding insulin levels (Figure 5B) and HOMA-IR (Figure 5C) in female offspring.

These findings suggest that CAF-induced maternal obesity affects glucose metabolism and promotes insulin resistance in the offspring in a sex-dependent manner. Opposed to what was observed in lipid metabolism, glucose disturbances appear to be more severe in males. On the other hand, CBD treatment was able to reduce plasma insulin in male offspring of obese dams to control levels, which led to an improved HOMA-IR in this group as well.

### 3.5. Cannabidiol treatment reverts LPS-induced endotoxemia caused by maternal obesity

**FIGURE 6:**
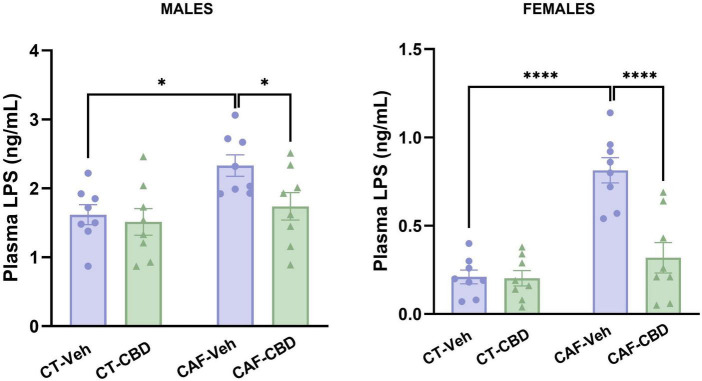
Plasma levels of lipopolysaccharides (LPS) of the offspring. Maternal obesity increased circulating LPS in both males and females, but cannabidiol (CBD) treatment was able to decrease its levels. Data are presented as mean ± SEM. *n* = 8–10/group. *p < 0.05 ^****^p < 0.0001.

Plasma levels of LPS were measured to evaluate metabolic endotoxemia. In male offspring, we found a maternal diet effect (*F*_1,28_ = 7.215; *p* = 0.0120). Male CAF-Veh showed a higher concentration of LPS than CT-Veh (*p* = 0.0148), while CAF-CBD had lower levels when compared to CAF-Veh (*p* = 0.0470) (Figure 6). In female offspring, there were maternal diet (*F*_1,28_ = 32.46; *p* < 0.0001) and treatment (*F*_1,28_ = 15.81; *p* = 0.0004) effects and an interaction between both (*F*_1,28_ = 14.88; *p* = 0.0006). Female CAF-Veh presented higher levels of plasma LPS than CT-Veh (*P* < 0.0001), while CAF-CBD had lower levels than CAF-Veh (*p* < 0.0001) (Figure 6). Thus, CBD treatment seems to be effective to reduce plasma levels of LPS in the offspring of obese dams.

### 3.6. Cannabidiol treatment rescues hypothalamic neuroinflammation resulted from maternal obesity

Real time quantitative polymerase chain reaction (RT-qPCR) was conducted to evaluate gene expression of TNFα, IL6, IL1β, GFAP, and IBA-1 in the hypothalamus.

**FIGURE 7:**
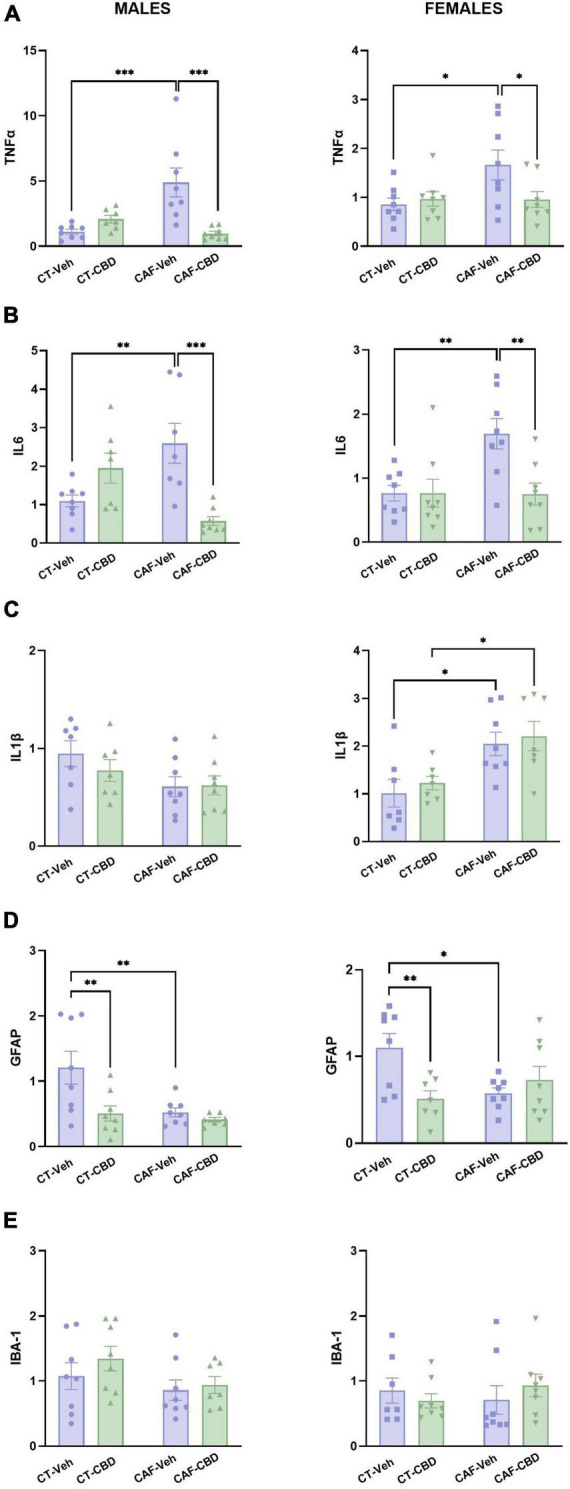
Relative gene expression of tumor necrosis factor-alpha (TNFα), interleukin 6 (IL6), interleukin 1β (IL1β), glial fibrillary acidic protein (GFAP), and ionized calcium-binding adapter molecule 1 (IBA1) in the hypothalamus of the offspring. Maternal obesity increased the expression of TNFα **(A)** and IL6 **(B)** in both males and females, with a positive effect of cannabidiol (CBD) treatment. Maternal obesity increased the expression of IL1β only in females, with no effect of CBD **(C)**. Both maternal obesity and CBD treatment decreased GFAP expression in males and females when compared to control diet (CT) **(D)**. No difference was found in IBA1 expression **(E)**. Data are presented as mean ± SEM. *n* = 6–8/group. *p < 0.05 ^**^p < 0.01 ^***^p < 0.001.

In male offspring, the gene expression of TNFα showed a maternal diet (*F*_1,27_ = 4.939; *p* = 0.0348) and CBD treatment (*F*_1,27_ = 6.035; *p* = 0.0207) effects, and also an interaction between both factors (*F*_1,27_ = 16.34; *p* = 0.0004). Male CAF-Veh showed higher levels of TNFα mRNA than CT-Veh (*p* = 0.0002), while CAF-CBD had lower levels than CAF-Veh (*p* = 0.0001) (Figure 7A). Regarding IL6 gene expression, there was an interaction between maternal diet and CBD treatment (*F*_1,26_ = 20.20; *p* = 0.0001). Male CAF-Veh showed higher levels of IL6 mRNA than CT-Veh (*p* = 0.0054), while CAF-CBD had lower levels than CAF-Veh (*p* = 0.0003) (Figure 7B). Regarding IL1β expression, there was a maternal diet effect (*F*_1,26_ = 4.919; *p* = 0.0355), with no differences among groups on the *post-hoc* test (Figure 7C). Regarding GFAP expression, there were maternal diet (*F*_1,27_ = 6.816; *p* = 0.0146) and CBD treatment (*F*_1,27_ = 7.402; *p* = 0.0113) effects. CAF-Veh presented much lower levels of GFAP mRNA than CT-Veh (*p* = 0.0055), while CT-CBD had lower levels than CT-Veh as well (*p* = 0.0045) (Figure 7D). There were no differences in IBA-1 expression (Figure 7E).

In female offspring, there was an interaction between maternal diet and CBD treatment (*F*_1,28_ = 4.250; *p* = 0.0486) regarding the gene expression of TNFα. Female CAF-Veh presented higher levels of TNFα mRNA than CT-Veh (*p* = 0.0156), while CAF-CBD had lower levels than CAF-Veh (*p* = 0.0351) (Figure 7A). Regarding IL6 expression, there was a maternal diet effect (*F*_1,28_ = 5.637; *p* = 0.0247), a treatment effect (*F*_1,28_ = 6.014; *p* = 0.0207) and an interaction (*F*_1,28_ = 6.057; *p* = 0.0203). CAF-Veh presented higher levels of IL6 mRNA than CT-Veh (*p* = 0.0039), while CAF-CBD had lower levels than CAF-Veh (*p* = 0.0034) (Figure 7B). There was a maternal diet effect (*F*_1,25_ = 15.62; *p* = 0.0006) on IL1β gene expression. Both CAF-Veh and CAF-CBD showed higher levels of IL1β mRNA than their CT (*p* = 0.0150 and *p* = 0.0255), with no effect of CBD treatment (Figure 7C). Regarding GFAP expression, there was an interaction between maternal diet and CBD treatment (*F*_1,27_ = 8.541; *p* = 0.0069). CAF-Veh showed lower levels of GFAP mRNA than CT-Veh (*p* = 0.0123), while CT-CBD had lower levels than CT-Veh as well (*p* = 0.0068) (Figure 7D). No differences were found in IBA-1 expression among groups (Figure 7E).

These findings indicate that maternal obesity leads to hypothalamic inflammation in the offspring. Nonetheless, treatment with CBD reduced the gene expression of the proinflammatory cytokines.

## 4. Discussion

Genetic and epidemiological studies provide evidence supporting the contribution of a transgenerational background of parental obesity to the development of obesity itself and further metabolic risks in the offspring (13, 46–48). Other than understanding the underlying mechanisms through which parental obesity takes its toll on the offspring’s health, an increasing body of research has been raising resolutive approaches. However, most of them rely on preventive measures targeted at the pre-conception and/or gestational period (49). Here, to address the problem once the damage is already set, we investigated the effects of CBD treatment on the offspring as a way to attenuate the negative outcomes of maternal obesity.

An increasing number of studies have addressed the pleiotropic role of the endocannabinoid system on metabolic regulation at the central and peripheral levels. Endocannabinoid regulation of metabolism is extremely relevant to the central nervous system (CNS), especially in the hypothalamus where it plays a pivotal role on energy balance and feeding behaviors, contributing not only for the pathogenicity of obesity but also the development of eating disorders (34, 36–38, 50, 51). Nonetheless its receptors are also expressed in peripheral organs such as the adipose tissue, liver, skeletal muscle, pancreas, kidney, and gastrointestinal tract (52), hence its particularly promising modulation in the context of obesity and metabolic disorders (53, 54). Here, we show that CBD treatment is able to revert a number of metabolic dysfunctions and neuroinflammation arising from maternal consumption of CAF during pregnancy and lactation, including higher visceral adiposity, insulin resistance, endotoxemia, and overexpression of inflammatory markers in the hypothalamus of the offspring.

Besides obesity itself, the consumption of a diet rich in saturated fats and carbohydrates is also associated with the development of metainflammation, a chronic and self-sustained state of low-grade inflammation (55). In this study, we demonstrated that both male and female offspring of obese mothers had higher visceral WAT deposits and fasting glucose levels, followed by elevated plasma cholesterol and triglycerides levels in females and insulin in males. Previous studies with different models of maternal obesity have established that both the gestation and the lactation-suckling periods are critical for WAT development, impacting epigenetic regulation of key genes for energy metabolism–such as dopamine and opioid genes related to food behavior (56) and hypothalamic nutrient sensors (46)–and altering long-term adiposity set points (57, 58). In a model of maternal high-fat diet (HFD), the offspring of obese mothers showed an increased expression and activity of stearoyl-CoA desaturase-1 (SCD1), a key enzyme of fatty acid (FA) metabolism. SCD1 converts saturated FAs, such as palmitate and stearate, to monounsaturated FAs, the predominant substrates for triglyceride synthesis (59). It is important to highlight that CAF, which closely mimics the Western urban eating patterns, is not only high in sugar but also saturated fats, with palmitate being the most predominant FA, hence the prominent impact of this dietary pattern on lipid profile (60, 61).

Interestingly, no effect of maternal obesity was found on total body weight, even though WAT deposition was altered. This finding could be due to a diminished muscle mass that may have compensated for the heavier adiposity. In previous studies, both 3- and 12-weeks old pups from CAF-fed mothers showed equal or lower body weight and lean mass but greater fat accumulation than controls, which has been described as the thin-outside-fat-inside phenotype (62, 63). Also, since in our study the offspring were fed a normal diet after weaning, we were able to show that the metabolic impairments observed were independent of the offspring’s own diet. However, in previous studies, when offspring of CAF-fed dams were given CAF after weaning, there was no increase in body weight at puberty (4 weeks of life), but animals had higher weight at adulthood (16 weeks of life), and no difference in visceral adiposity was reported in male offspring. Thus, differences in body composition seem to be dependent on the post-weaning diet as well as the sex of the offspring (64, 65).

Cannabidiol (CBD) treatment was able to mitigate most of the metabolic dysfunctions caused by maternal obesity, reducing visceral fat content and IR in males, plasma triglyceride levels in females, and plasma LPS in both sexes. CB1 receptor activation is generally considered a powerful orexigenic signal; thus, the endocannabinoid system’s inhibition is beneficial for treating obesity and related metabolic diseases. Since CBD is an allosteric modulator of CB1 receptors, inhibiting its activation by endogenous ligands or exogenous agonists might trace the pathway through which CBD attenuates peripheral disturbances arising from maternal obesity (66). CB1-KO mice maintained on a normocaloric, standard diet have been shown to have a decreased body weight gain over time, which was associated with increased energy expenditure and elevated β(3)-adrenergic receptor and uncoupling protein-1 (UCP1) mRNA levels in the brown adipose tissue, suggestive of enhanced peripheral sympathetic activation and thermogenesis (66).

Diets high in saturated FAs, such as the Western diet, increase the uptake and storage of sphingolipids and their essential fractions, such as ceramide, sphingosine, sphinganine, and sphingomyelin. Interestingly, previous data suggest that phytocannabinoids and other agonists of CB1 or CB2 receptors can modulate sphingolipid concentrations in specific organs under the increased availability of FAs in the diet. CBD significantly lowered the concentration of sphingolipids in the adipose tissue (67), the skeletal muscle (31), and the brain by increasing catabolism, inhibiting salvage and/or *de novo* synthesis, which restores the tissue’s insulin sensitivity and, therefore, attenuates IR (68). Other than that, other mechanisms are proposed for the beneficial effects of CBD on metabolic disorders in peripheral organs, such as the protective effect of CBD on adipose-derived stem cells against endoplasmic reticulum stress and its complications related to IR and diabetes (69) and attenuation of oxidative stress and inflammatory response, associated with an improved n-6/n-3 polyunsaturated fatty acids (PUFAs) ratio in the white and red skeletal muscle (70), indicating a narrow relationship between the endocannabinoid system and hormonal and energetic balance.

It is worth noting that the sexual dimorphisms observed in this study regarding glucose metabolism and IR corroborate with what has been demonstrated in different models of obesity and maternal obesity (71). Female sex hormones play a fundamental role in dimorphic insulin signaling since estrogens increase insulin sensitivity in metabolic tissues and upregulate insulin transcription in pancreatic beta cells as well as GLUT4 synthesis in adipose tissue and muscle (72). Female mice fed an HFD showed reduced susceptibility to developing obesity-induced IR and WAT inflammation when compared to HFD-fed males. Meanwhile, HFD-fed males treated with estradiol presented the same protective effect as females, indicating that the dimorphic effects of obesity on IR may be due to estrogen-mediated reductions in WAT inflammation (73). Furthermore, a recent study has shown that an androgen-driven gut microbiome may also be responsible for the increased susceptibility to IR in males since gut microbiome depletion abolishes sex-biased glucose metabolism in HFD-fed mice (74).

A growing body of evidence suggests that males are more sensitive to intrauterine hyperglycemia as well; hence both animal and human studies show the same pattern of higher risk for obesity and IR in male offspring of obese/diabetic mothers. In a model of maternal high-sucrose diet (HSD), female HSD offspring were shown to be more glucose intolerant, while male counterparts were more insulin resistant (75). Furthermore, human cohort and prospective studies have shown a strong correlation between offspring metabolic impairments and maternal diabetes for males but not for females (76–78). This relationship may be explained by the fact that, during preimplantation, the male embryo is believed to have a greater ability to adapt to the adverse environment and, as a result, has a higher sensitivity to programming influences (79).

In addition, our results showed that CBD effectively reverted the increase in plasma LPS levels in both male and female CAF offspring. Obesogenic diets change the gut microbiota composition by altering the Firmicutes: Bacteroidetes ratio, the two most detected bacterial phyla in rodents as well as humans. In normal-weight animals this relation is characterized by a high ratio of Bacteroidetes to Firmicutes, while the opposite is found in obese counterparts (80, 81). It has been proposed that Firmicutes bacteria are more effective in extracting energy from food than Bacteroidetes, thus promoting a more efficient absorption of calories and boosting weight gain (82, 83). This imbalance resulting from obesogenic diets can be traced back to the overabundance of refined sugars and fats as well as the low intake of vegetables, fruits and dietary fibers (84–87). The simultaneous collapse of the gut barrier with increased permeability allows high levels of LPS, Gram negative bacteria’s most potent immunogenic component, to reach the bloodstream and initiate a diffuse inflammatory process named endotoxemia (88). The structural components of LPS are recognized by B cells *via* cluster of differentiation 14 (CD14) and toll-like receptor 4 (TLR4), thus leading to nuclear factor kappa-B (NFkB) activation and the release of pro-inflammatory cytokines, such as TNFα and IL1β (89). During pregnancy, these pro-inflammatory mediators, together with LPS itself, can interact with the placenta and cause a range of disturbances, including immature blood vessels, hypoxia, increased inflammation, autophagy, and altered stress markers (90, 91). In that sense, several models of maternal immune activation rely on prenatal exposure to LPS, resulting in a myriad of altered physiological and neurological outcomes in the offspring (92, 93). The data seen here indicates that the endotoxemia caused by obesogenic diets affects not only the pregnant mother but can be seen in the offspring later on, independently of the offspring’s own diet. These findings corroborate with previous studies demonstrating that maternal obesity during gestation and/or lactation negatively impacts the offspring’s gut microbiota (94). On the other hand, CBD has rescued this damage on the offspring, lowering plasma LPS to control levels. Even though we have not performed gut-specific analysis, previous studies in different pre-clinical and clinical models lead us to infer that the effects observed may be due to the influence of CBD on gut microbiota composition (95–97) and/or a protective effect on maintaining gut barrier integrity (98–102).

Regarding the alterations provoked by maternal overnutrition on CNS neuroinflammation, here we show that the treatment with CBD was able to rescue hypothalamic inflammation by reducing gene expression of TNFα and IL6 in the offspring of obese dams. The hypothalamus is one of the main homeostatic centers of the CNS and, therefore, needs to be effectively responsive to fluctuations in peripheral systems. However, due to its naturally increased permeability in order to better receive and respond to signals coming from metabolic organs, the hypothalamus is also one of the first brain regions to suffer with systemic disruptions, resulting in neuroinflammation (103, 104). In the present study, we have demonstrated molecular alterations that are trademarks of neuroinflammation in the hypothalamus of the offspring born from CAF-fed dams. In both sexes, the expressions of TNFα and IL6 were increased in CAF-Veh animals, while the expression of IL1β was increased only in females. These findings corroborate with previous data from a different model of maternal obesity that demonstrates that mice born from mothers fed a HFD diet have increased expression of these inflammatory markers in the hypothalamus compared to the offspring of lean parents (105).

Tumor necrosis factor-alpha (TNFα), IL6, and IL1β are well-known pro-inflammatory cytokines involved in microglial and astrocytic activation in the entire nervous tissue. However, especially in the hypothalamus, they have a remarkable role in the modulation of hypothalamic feeding circuits. It has been previously demonstrated that HFD and high-carbohydrate diets stimulate orexigenic neuropeptide Y/agouti-related peptide (NPY/AgRP) neurons to produce advanced glycation end products, which activate TNFα, enhancing microglia reactivity. This scenario results in the dysfunction of anorexigenic neurons, altering the appetite-regulatory circuits (106). In addition, Proopiomelanocortin (POMC) neurons, which present anorexigenic activities, also suffer a significant impact from maternal obesity (107). The melanocortin system plays an important role on the regulation of appetite, energy expenditure, and metabolism, therefore, impairments in the POMC and melanocortin 4 receptor (MC4R) pre- and post-translational processing are forerunners for the development of obesity (108, 109). Decreased activity in POMC cells has been shown to be associated with increased food intake and obesity (107) and has been demonstrated in the offspring of obese mothers (110–113). When observing the precise localization of NPY and POMC in the hypothalamus of the offspring of obese mothers, Ornellas and collaborators found that NPY nerve fibers from the ARC to the periventricular nucleus and around the third ventricle were increased, while POMC were diminished in the same areas (105). Variations in the reactivity and/or distribution of hypothalamic astrocytes also seem to affect synaptic organization and POMC responsiveness to glucose, which is associated with energy and metabolic imbalances (114, 115).

Although endocannabinoid signaling has been implicated in the modulation of both food intake and energy expenditure, a complete understanding of its role in the hypothalamus is still lacking. A recent study demonstrated that a HFD diet in CB1 receptor-deficient mice contributes to the offspring’s nutritional programming, resulting in increased susceptibility to metabolic challenges both perinatally and during adulthood (116). Additionally, maternal HFD has been shown to upregulate CB1 hypothalamic expression in the offspring, which was associated with leptin pathway impairment and increased susceptibility to obesity (117–119). Other than that, few studies have evaluated cannabinoid modulation in the context of parental obesity, however, the findings shown here are still in line with different models that show the anti-inflammatory effects of CBD on other neuroinflammatory conditions (95, 120–122). Elevated hypothalamic endocannabinoid content has been associated with higher orexigenic signaling of ghrelin (123–125) and defective leptin signaling, observed in genetic models of obesity such as obese Zucker rats and *db/db* and *ob/ob* mice (126, 127). These findings suggest that endocannabinoid mediators contribute to hyperphagia and obesity, which also supports the restorative effects of CBD treatment, once it reduces endocannabinoid signaling, especially through CB1 receptors (128). When it comes to inflammation, effects of CBD *via* CB2 receptor are more distinguished, since this receptor is more predominantly expressed on immune cells, including glial cells. CB2 expression is upregulated in microglia stimulated with pro-inflammatory cytokines, indicating a significant role of CB2 in the regulation of neuroinflammatory states (129). In line with this, CBD has been shown to exert a CB2-dependant anti-inflammatory effect on microglial inflammation (23, 130).

Astrogliosis is a very well-established marker for obesity-related neuroinflammation (16, 131). Variations in the reactivity and/or distribution of hypothalamic astrocytes seem to affect synaptic organization and responsiveness to peripheral fluctuations, which is associated with energy and metabolic imbalances (114, 115). In animal models of obesity, gene and protein expression of the glial fibrillary acidic protein (GFAP), an astrocyte marker, are commonly higher in obese groups when compared to control animals (131–134). Interestingly, we have found that GFAP gene expression was reduced in the hypothalamus of the offspring of obese mothers. This result may have been induced by an adaptative reprogramming mechanism in response to the exposure to a harmful intrauterine environment during neurodevelopment, indicating that molecular mechanisms that rule maternal obesity-induced neuroinflammation may differ from the ones associated with obesity in the individual itself (135–137). Reduction in astrocyte expression can be deleterious during neurodevelopment since these cells play a pivotal role in synapse maturation, and their reduced expression is related to a range of neurological disorders (138–140). We have observed the same reduction of GFAP expression in CBD-treated CT offspring, however, we cannot affirm that the same detrimental effect applies. The reduction in GFAP expression of CAF offspring is a response to a prenatal immune challenge, while the reduction seen in CT-CBD is more likely to be the result of the anti-inflammatory activity of CBD (141, 142).

Regarding microglial activation, unlike previous studies (143, 144), we have not found any differences in the gene expression of ionized calcium-binding adapter molecule 1 (IBA1) in the hypothalamus of the offspring of obese mothers. However, the expression of IBA1 is related to the proliferation and distribution of microglial cells and not the polarization toward a pro-inflammatory state (145). Furthermore, it has recently been described that prenatal immune stress blunts microglia reactivity throughout life (146), which means that the expression levels of microglial cells may remain at control levels, but their innate reactivity to immune stressors can be defective.

These gene expression patterns are consistent with impaired energy and metabolic regulation in the hypothalamus, which might have originated the peripheral deficits observed in the offspring of obese mothers. Together, these results indicate an intricate interplay between peripheral and central counterparts in both the pathogenicity of maternal obesity and the modulation of the endocannabinoid system by CBD. In this context, the impairment of internal hypothalamic circuitry caused by neuroinflammation runs in tandem with the disruptions of important metabolic processes, which can be attenuated by CBD treatment in both ends.

## Data availability statement

The raw data supporting the conclusions of this article will be made available by the authors, without undue reservation.

## Ethics statement

The animal study was reviewed and approved by Comissão de Ética no Uso de Animais–Universidade Federal de Ciências da Saúde de Porto Alegre.

## Author contributions

FR, MG, and RG: conceptualization. FR, JJ, GF, VD, SE, and TD: *in vivo* experimental procedures and *ex vivo* tissue and sample analysis. FR, JJ, and RG: statistical analysis. MG and RG: critical revision of the manuscript. All authors have writing, read, edited, and approved the final version of the manuscript.
